# Charge-Shift Bonding in Xenon Hydrides: An NBO/NRT Investigation on HXeY···HX (Y = Cl, Br, I; X = OH, Cl, Br, I, CCH, CN) via H-Xe Blue-Shift Phenomena

**DOI:** 10.3389/fchem.2020.00277

**Published:** 2020-04-23

**Authors:** Guiqiu Zhang, Yue Su, Xiaoran Zou, Lei Fu, Junjie Song, Dezhan Chen, Chuanzhi Sun

**Affiliations:** Key Laboratory of Molecular and Nano Probes, College of Chemistry, Chemical Engineering and Materials Science, Collaborative Innovation Center of Functionalized Probes for Chemical Imaging, Ministry of Education, Shandong Normal University, Jinan, China

**Keywords:** chemical bonding, charge-shift bonds, electron-pair bonds, resonance bonding, noble-gas hydride, hypervalent molecule, blue shifts, NBO/NRT methods

## Abstract

Noble-gas bonding represents curiosity. Some xenon hydrides, such as HXeY (Y = Cl, Br, I) and their hydrogen-bonded complexes HXeY···HX (Y = Cl, Br, I; X = OH, Cl, Br, I, CN, CCH), have been identified in matrixes by observing H-Xe frequencies or its monomer-to-complex blue shifts. However, the H-Xe bonding in HXeY is not yet completely understood. Previous theoretical studies provide two answers. The first one holds that it is a classical covalent bond, based on a single ionic structure H-Xe^+^ Y^−^. The second one holds that it is resonance bonding between H-Xe^+^ Y^−^ and H^−^ Xe^+^-Y. This study investigates the H-Xe bonding, via unusual blue-shifted phenomena, combined with some NBO/NRT calculations for chosen hydrogen-bonded complexes HXeY···HX (Y = Cl, Br, I; X = OH, Cl, Br, I, CN, CCH). This study provides new insights into the H-Xe bonding in HXeY. The H-Xe bond in HXeY is not a classical covalent bond. It is a charge-shift (CS) bond, a new class of electron-pair bonds, which is proposed by Shaik and Hiberty et al. The unusual blue shift in studied hydrogen-bonded complexes is its H-Xe CS bonding character in IR spectroscopy. It is expected that these studies on the H-Xe bonding and its IR spectroscopic property might assist the chemical community in accepting this new-class electron-pair bond concept.

## Introduction

The chemical bond is the most central concept in chemistry. The model of chemical bonding can help chemists to understand and design matter. Despite the apparent utility of the model of chemical bonding, it is incomplete. Developing bonding models is undoubtedly important to our understanding of novel molecules, such as the molecules of noble gas chemistry (Grandinetti, 2018).

The challenge to noble-gas bonding comes mainly from the inertness of noble-gas atoms. However, significant progress has been made in noble-gas chemistry during the past 20 years. Besides noble-gas hydrides (Khriachtchev et al., 2009), noble gas–noble metal complexes have been studied for their thermodynamic and kinetic stabilities in theory (Jana et al., 2017, 2018a,b; Pan et al., 2019; Saha et al., 2019). Experimentally, about 30 noble-gas hydrides had been identified by the beginning of 1995 (Pettersson et al., 1995; Räsänen et al., 2000; Lundell et al., 2002; Feldman et al., 2003; Khriachtchev et al., 2003, 2009; Duarte and Khriachtchev, 2017). About 10 hydrogen-bonded complexes between HNgY (Ng = Xe, Kr) and H_2_O/HCl/HBr/HI/HCCH have been well-characterized via infrared (IR) spectroscopy. Interestingly, these complexes show unusual shifts of the H-Xe stretching vibration. For example, the H-Xe stretching mode of the complex HXeI**···**HCCH exhibits a blue shift of 49 cm^−1^, in comparison with the monomer HXeI (Zhu et al., 2015). Some other complexes such as HXeBr**···**H_2_O and HXeI**···**H_2_O are characterized by much larger experimental blue shifts of the H-Xe stretching frequency (>100 cm^−1^) (Tsuge et al., 2014). The largest blue shift (300 cm^−1^) has been reported for the H-Kr stretching mode of the complex HKrCl**···**HCl (Corani et al., 2009). These experimental findings provide theoretical chemistry researchers with excellent opportunities to develop a bonding model for noble-gas hydrides.

In pioneering theoretical work, Last and George put forward one simple ionic structure model H-Ng^+^*Y*^−^ (Last and George, 1988), where H-Ng^+^ belongs to a classical covalent bond, to be exact, an electron-sharing bond, while the interaction between H-Ng^+^ and Y^−^ comes from electrostatic attraction. Energy decomposition analyses (EDAs) carried out by Frenking for HArF provided a consistent bonding picture (Lein et al., 2004). Besides this ionic structure model, empirical resonance bonding models were proposed by Räsänen's group (Pettersson et al., 1999) and Alabugin's group (Alabugin et al., 2004). Recently, our group has carried out the resonance bonding analyses for noble-gas hydrides (Zhang et al., 2016) by using Natural Bond Orbital (NBO) and Natural Resonance Theory (NRT) methods (Glendening and Weinhold, 1998a,b; Glendening et al., 1998, 2001, 2013a,b; Weinhold and Klein, 2014; Weinhold et al., 2016). We found that each molecule HNgY could be best described as three structures, H-Xe^+^*Y*^−^, *H*^−^*Xe*^+^-Y, and H^∧^Y, where the first two resonance structures mix to form hyperbonding of H-Xe^+^*Y*^−^↔ H^−^*Xe*^+^-Y, as proposed by Weinhold and Landis (2005b). Such bonding provides a picture of resonance covalency for noble-gas hydrides (Weinhold and Klein, 2012, 2014; Landis and Weinhold, 2013; Zhang et al., 2015, 2017a; Jiao and Weinhold, 2019).

It is important to note that they are two variants of covalent bonding, which are related to two kinds of electron-pair bonds. They are our familiar electron-sharing bonds and dative bonds. The difference between these two types of bonds is due to the origin of the bonding electrons. In electron-sharing bonds, each fragment provides one electron. In dative bonds, both electrons come from one fragment, which donates two electrons to the vacant orbital of the other. Also note that besides our familiar electron-sharing bonds and dative bonds there is one new kind of electron-pair bonds, charge-shift (CS) bonds. The concept of charge-shift bonds was first proposed by Shaik and Hiberty et al. in 1992, to describe a new-class electron-pair bonds, such as F_2_ (Shaik et al., 1992). Ten years later, they further presented experimental manifestations of CS bonding (Shaik et al., 2005, 2009). They have applied the concept of CS bonds to the understanding of bonding and stabilities of several hypervalent molecules (Braïda and Hiberty, 2013; Braïda et al., 2014), such as XeF_2_. In 2018, Grandinetti pointed out that a stabilization like HNgY is, generally, known as CS bonding (Grandinetti, 2018). Indeed, the decades following their original work saw more CS bonding molecules. Very recently, they published their latest review on CS bonds (Shaik et al., 2020).

Given that XeF_2_ and HXeY are similar in geometrical and electronic structures, two obvious questions arise: (1) Is the H-Xe bond in HXeY a charge-shift bond? (2) What is the H-Xe CS bonding character in IR spectroscopy? This study explores these two questions through blue-shifted phenomena, with the help of NBO/NRT analyses. We choose HXeY···HX (Y = Cl, Br, I; X = OH, Cl, Br, I, CN, CCH) as study systems. Some of them are identified in the matrix experiment. We analyze the relationship between the H-Xe bonding and H-Xe blue shifts. We aim to understand the H-Xe bonding in HXeY and to extend CS bonding concepts to noble-gas hydrides.

This paper will be organized as follows. Firstly, we summarize the computational details and discuss the geometrical structural details and H-Xe IR spectroscopic properties for our studied monomers and complexes. Secondly, we analyze the resonance bonding of HXeY, especially for the bonding between H and Xe. Thirdly, we analyze the H-Xe bond order. And we determine that the H-Xe bond order must include the contributions of two resonance structures. Fourthly, we confirm that the H-Xe in HXeY is a charge-shift bond, and further analyze its CS bonding character. Finally, we present the concluding summary, with emphasis on the H-Xe CS bonding and its bonding character.

## Computational Details

The geometry optimization and vibrational frequency calculations were carried out with the Gaussian 09 program at the level of the second order MØller-Plesset perturbation theory (MP2) (Head-Gordon et al., 1988; Frisch et al., 2009). The def2-TZVPPD basis set was used. This basis set, taken from the EMSL basis set library (Feller, 1996; Schuchardt et al., 2007), is the triple-zeta-valence basis set augmented with two sets of polarization and diffuse basis functions. No imaginary frequencies were found in any case, which confirms that our obtained structures are true local minima on the potential energy surface. The NBO and NRT were employed to analyze the bonding of our studied systems with the NBOPro 6.0 program (Glendening and Weinhold, 1998a,b; Weinhold, 2012, 2013). Directed NBO analyses could provide the second order perturbation energy of a donor-acceptor interaction in the best Lewis structure. For any other resonance structure, we use $CHOOSE keylist to calculate the second order perturbation energy of a donor-acceptor interaction. It is important to attach the $NRTSTR keylist in NRT analyses, for it insures a consistent set of reference structures for NRT comparisons of studied complexes. The NBO-based natural resonance theory complements and extends NBO analyses to other resonance structures. By using $NRTSTR keylist to specify key structures as reference structures, we can obtain accurate weightings (ω_I_, ω_II_, ω_III_…) of resonance structures, and NRT bond orders (*b*_AB_) that express the strength of resonance-weighted chemical bonds between any atom pair. More importantly, the NBO/NRT-based models provide a framework for analyzing chemical bonding in terms of familiar concepts, such as Lewis structures, resonance, and donor-acceptor interactions. Herein, we use NBO/NRT methods to analyze H-Xe bonding. Besides, the NBOview 2.0 module was acquired to obtain the orbital overlap graphics.

## Results and Discussions

### Geometry and Blue Shifts

**Figure 1:**
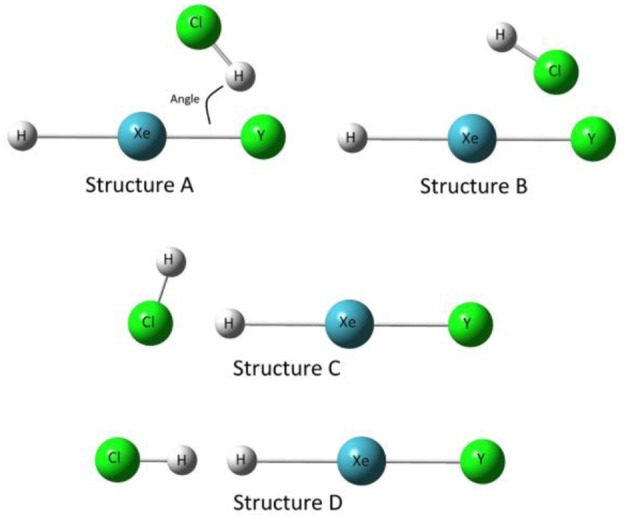
Four structures of complexes HXeY···HCl (Y = Cl, Br, I).

In general, four structures need to be considered for each of the complexes HXeY···HX (Y = Cl, Br, I; X = OH, Cl, Br, I, CN, CCH). Take HXeY···HCl, as shown in Figure 1, as one representative example. In the first two structures (A and B), the HCl moiety is closed to the halogen atom of the monomer HXeY to form a bent structure. Structure A is stabilized with the Cl-H**···**Y hydrogen bond, whereas Structure B is dependent on the Xe-Y**···**Cl halogen bond. Structure C, whose halogen atom in the moiety is collinear with the monomer HXeY, is formed by the Cl atom interacting with the H atom. Structure D is stabilized with the dihydrogen Cl-H**···**H-Xe bond with all atoms in line. Experimentally, it has been observed that several infrared absorption bands originate from the H-Xe stretching mode for our chosen complexes. With the aid of quantum chemical calculations, they are assigned to Structure A (Lignell et al., 2008; Tsuge et al., 2013, 2014; Zhu et al., 2015). Therefore, the following analyses are restricted to Structure A.

As shown in Structure A in Figure 1, HXeY maintains its original linear structure for all the HXeY···HX (Y = Cl, Br, I; X = OH, Cl, Br, I, CN, CCH) optimized geometry. At the MP2/def2-TZVPPD level, the calculated H-Xe and Xe-Y bond lengths (*R*_H−Xe_, *R*_Xe−Y_) and vibrational frequency shifts of H-Xe stretching mode in complexes HXeY···HX (Y = Cl, Br, I; X = OH, Cl, Br, I, CN, CCH) as well as in monomers HXeY are collected in Table S1 and compared with the available experimental data. Note that Räsänen's group has computed *R*_H−Xe_ and *R*_Xe−Y_ as well as a variety of H-Xe frequency blue shifts for some of the complexes HXeY···HX (Y = Cl, Br, I; X = OH, Cl, Br, I, CN, CCH) at the CCSD(T) level. The difference in calculated H-Xe bond lengths at both MP2 and CCSD(T) levels is <0.060 Å, whereas for Xe-Y bond lengths the largest difference is 0.048 Å in the monomer HXeI. These comparisons show that the MP2/def2-TZVPPD is an appropriate model chemistry for this study. Thus, the following discussion will be based on the calculated data at the MP2/def2-TZVPPD level.

Data in Table S1 show that the complexation has non-negligible influences on monomers HXeY. On the one hand, the bond lengths of H-Xe in complexes become shorter as compared with the value in the corresponding HXeY monomer. Taking HXeCl species as one example, H-Xe bond lengths *R*_H−Xe_ in complexes are 1.646, 1.645, 1.644, 1.646, 1.648, and 1.655 Å for HX = H_2_O/HCl/HBr/HI/HCN/HCCH, respectively, while the H-Xe bond length of the monomer HXeCl is 1.666 Å. This indicates that the interaction between H and Xe atoms in monomers HXeY is weaker than that in corresponding complexes HXeY···HX (Y = Cl, Br, I; X = OH, Cl, Br, I, CN, CCH). On the contrary, the bond lengths of Xe-Y within complexes are slightly larger than that in the corresponding monomer HXeY, which means that the interaction between Xe and Y atoms is stronger in monomers than in corresponding complexes. It is worthwhile noting that the calculated *R*_H−Xe_ ranges from 1.644 to 1.708 Å, slightly larger than the covalent limits *R*_cov_[*r*_cov_(H)+*r*_cov_(Xe)] for 1.63 Å and significantly shorter than VdW limits *R*_vdw_[*r*_vdw_(H)+*r*_vdw_(Xe)] for 3.86 Å (Pyykkö, 2015; Rahm et al., 2016). This indicates strong covalency of the H-Xe bond.

On the other hand, the complexation leads to experimentally observable blue shifts of the H-Xe stretching frequency, which seems to be normal phenomena according to Khriachtchev's group studies. The calculated and experimental vibrational frequency shifts of the H-Xe stretching mode are also collected in Table S1. Taking hydrogen-bonded complexes HXeCl···H_2_O, HXeBr···H_2_O and HXeI···H_2_O as one example group, calculations at the MP2 level predict blue shifts of 101, 116, and 139 cm^−1^, respectively. The experimental data are 82, 101, and 138 cm^−1^, respectively. Obviously, the calculated complexation-induced spectroscopic shift of the H-Xe stretching mode at the MP2 level is in qualitative agreement with the experimental values. These preliminary structural and blue-shifted analyses provide the backdrop for the following exploration of H-Xe bonding for Xe hydrides.

### H-Xe Resonance Bonding in HXeY

Earlier studies have found that the NBO/NRT method is a helpful tool to explore the resonance bonding because NBO analyses can provide the best Natural Lewis Structure (NLS), identify donor-acceptor orbital interactions, and estimate the second-order perturbation energy [*E*^(2)^] of each donor-acceptor orbital interaction (Glendening et al., 2013a,b; Weinhold, 2013; Weinhold et al., 2016).

**Figure 2:**
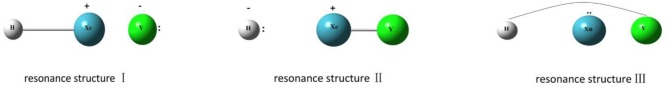
Three resonance structures for HXeY. A pair of dots represents a lone pair.

**Figure 3:**
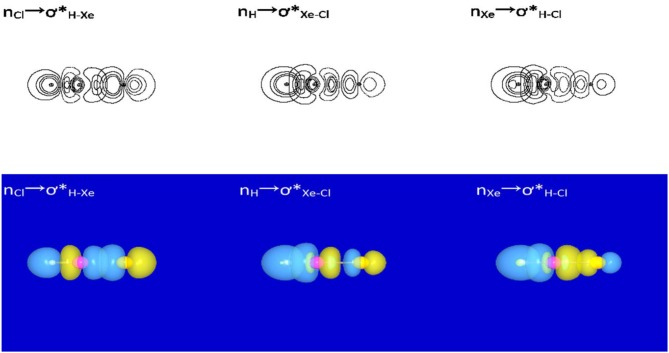
NBO orbital contour diagrams and 3-D surface views of donor-acceptor interactions for HXeCl.

Our studies begin with NBO/NRT analyses for HXeY species. The results show that each of the studied HXeY could be better described as a hybrid of the three structures (I,II,and III) as shown in Figure 2, where the H-Xe^+^:Y^−^ (I) is the best NLS. Table S2 lists three types of donor-acceptor interactions and the value of the second-order perturbation energies [*E*^(2)^] of the studied HXeY. For the best NLS H-Xe^+^:Y^−^ (I), the donor-acceptor interaction (${\text{n}}_{\text{Y}}\to \sigma_{\text{H}-\text{Xe}}^{*}$) takes place between the lone pair orbital of Y (n_Y_) and the antibonding orbital of H-Xe moiety ($\sigma_{\text{H}-\text{Xe}}^{*}$). Pay particular attention to such a delocalization interaction. It represents resonance mixing between H-Xe^+^:Y^−^ (I) and H:^−^ Xe^+^-Y (II), where the latter corresponds to the lone pair of H atom (n_H_) delocalizing to the antibonding orbital of Xe-Y moiety ($\sigma_{\text{Xe}-\text{Y}}^{*}$). As proposed by Weinhold et al., these two structures make up resonance bonding. Additionally, there is a non-negligible long-bonding structure H^∧^Y (III), shown in the final entry in Figure 2. It arises from the delocalization of Xe atom lone pair (n_Xe_) to the antibonding orbital of H-Y moiety ($\sigma_{\text{H}-\text{Y}}^{*}$). Special attention is paid to the unusual values of *E*^(2)^ for ${\text{n}}_{\text{Xe}}\to \sigma_{\text{H}-\text{Y}}^{*}$ and ${\text{n}}_{\text{H}}\to \sigma_{\text{Xe}-\text{Y}}^{*}$ interactions. Such results show that they are no longer suitable for the description of low-order perturbative NLS limit. Even in the unavailable value of some second-order perturbation energies, the importance of these three donor-acceptor interactions can be exhibited by the orbital overlap contour diagrams or 3-D surface views. Figure 3 presents one illustrative example. These results are consistent with our previous studies for HXeY noble-gas hydrides (Y = Cl, Br, I) at the B3LYP level of theory (Zhang et al., 2016).

Once again, the importance of resonance bonding H-Xe^+^ Y^−^ ↔ H^−^ Xe^+^-Y is emphasized. On the one hand, resonance bonding is an essential feature of H-Xe bonding in HXeY. On the other hand, we note that earlier studies on the bonding of noble-gas hydrides have already analyzed the leading resonance structure H-Xe^+^ Y^−^ (Pérez-Peralta et al., 2009; Juarez et al., 2011). In the next section, we will carry out detailed analyses on H^−^ Xe^+^-Y, as well as H-Xe^+^ Y^−^.

### H-Xe Bond in HXeY Is Not a Classical Covalent Bond

**Figure 4:**
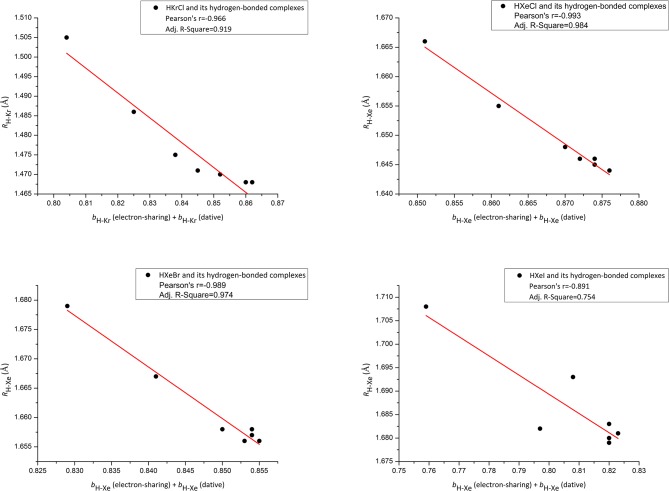
Correlation plot for the sum of electron-sharing and dative H-Xe/Kr bond orders [*b*_H−Xe/Kr_ (electron-sharing) + *b*_H−Xe/Kr_ (dative)]–H-Xe/Kr bond lengths (*R*_H−Xe/Kr_).

HXeY is a particularly simple and interesting molecule. Its H-Xe stretching modes provide experimental probes to learn more about the bonding of noble-gas hydrides. It was reported that the H-Xe stretching vibration frequencies of HXeY species are blue shifts upon complexation. In this section, we will explore the H-Xe bonding via this complexation effect, combining with quantitative NRT analyses on chosen hydrogen-bonded complexes. Table 1 displays the weighting of three resonance structures upon complexation for Xe cases. Obviously, the moiety H_2_O/HCl/HBr/HI/HCN/HCCH complexing with HXeY has a significant influence on the weighting of three resonance structures for HXeY. To be specific, for the long-bonding structure (Weinhold et al., 2016; Zhang et al., 2017b, 2018), its weighting always decreases upon complexation. This indicates that the complexation of the moiety H_2_O/HCl/HBr/HI/HCN/HCCH studied here is not beneficial to the stability of long-bonding in HXeY. For two other resonance structures, the ω_I_ decreases while ω_II_ increases relative to the corresponding monomer in studied hydrogen-bonded complexes except for the complex HXeCl**···**HCCH. For example, the weightings of H-Xe^+^:I^−^ and H:^−^ Xe^+^-I in the hydrogen-bonded complex HXeI**···**HI are 62.8 and 19.2%, compared with 66.4 and 9.5% in the monomer HXeI. In contrast, the complex HXeCl**···**HCCH shows a different trend. As it is easily seen, the weightings of H-Xe^+^:Cl^−^ and H:^−^ Xe^+^-Cl structures in the monomer HXeCl are 74.3 and 10.9% respectively; in the complex HXeCl**···**HCCH they are 76.1 and 9.7%, respectively. In short, for all of our studied complexes, the HXeY complexation with small molecules always leads to a decrease of the weighting about the long-bonding structure. For weightings of these two other resonance structures, one structure always shows a decreasing trend while the other exhibits an increasing trend for all of the studied complexes. In most of the Xe cases studied here, the complexation results in an increasing weighting of the resonance structure H-Xe^+^:Cl^−^, with a decreasing weighting of the resonance structure H:^−^ Xe^+^-Cl. In HXeCl**···**HCCH the situation is different. An opposite trend is seen for the complex HXeCl**···**HCCH. From preliminary NBO analyses on our studied complexes, peculiarities in HCCH complexes shown here, in Tables 1–**3**, and Figure 4, may be due to n_Xe(d)_ → $\pi_{\text{C}-\text{C}}^{*}$ donor-acceptor interaction.

**Table 1:** The weighting and its change of three resonance structures (H-Xe^+^ :Y^−^, H:^−^ Xe^+^-Y, H^∧^Y) upon HXeY complexation with H_2_O/HCl/HBr/HI/HCN/HCCH, compared with the monomer HXeY.

**Monomers/complexes**	**ω_I_**	**ω_II_**	**ω_III_**	**Sum**
HXeCl	74.3%	10.9%	14.9%	100.1%
HXeCl···H_2_O	70.3% (−4.0%)	16.4% (+5.5%)	12.7% (−2.2%)	99.4%
HXeCl···HCl	71.8% (−2.5%)	15.6% (+4.7%)	12.2% (−2.7%)	99.6%
HXeCl···HBr	72.3% (−2.0%)	15.3% (+4.4%)	12.0% (−2.9%)	99.6%
HXeCl···HI	72.0% (−2.3%)	15.4% (+4.5%)	12.1% (−2.8%)	99.5%
HXeCl···HCN	69.9% (−4.4%)	16.9% (+6.0%)	13.1% (−1.8%)	99.8%
HXeCl···HCCH	76.1% (+1.8%)	9.7% (−1.2%)	13.8% (−1.1%)	99.6%
HXeBr	71.3%	11.6%	17.1%	100.0%
HXeBr···H_2_O	67.0% (−4.3%)	17.8% (+6.2%)	14.6% (−2.5%)	99.4%
HXeBr···HCl	68.2% (−3.1%)	17.2% (+5.6%)	14.3% (−2.8%)	99.7%
HXeBr···HBr	68.7% (−2.6%)	16.8% (+5.2%)	13.9% (−3.2%)	99.4%
HXeBr···HI	68.4% (−2.9%)	17.0% (+5.4%)	14.1% (−3.0%)	99.5%
HXeBr···HCN	66.4% (−4.9%)	18.2% (+6.6%)	15.0% (−2.1%)	99.6%
HXeBr···HCCH	64.3% (−7.0%)	19.4% (+7.8%)	16.0% (−1.1%)	99.7%
HXeI	66.4%	9.5%	24.1%	100.0%
HXeI···H_2_O	61.6% (−4.8%)	20.3% (+10.8%)	18.1% (−6.0%)	100.0%
HXeI···HCl	62.5% (−3.9%)	17.1% (+7.6%)	20.0% (−4.1%)	99.6%
HXeI···HBr	63.1% (−3.3%)	19.2% (+9.7%)	17.2% (−6.9%)	99.5%
HXeI···HI	62.8% (−3.6%)	19.2% (+9.7%)	17.3% (−6.8%)	99.3%
HXeI···HCN	61.4% (−5.0%)	20.3% (+10.8%)	18.1% (−6.0%)	99.8%
HXeI···HCCH	59.0% (−7.4%)	21.6% (+12.1%)	19.3% (−4.8%)	99.9%

*Note that the ω_I_ in this table represents the sum of the weightings for the structures closely related with the best NLS H-Xe^+^ :Y^−^. Taking HXeCl···HI complex as one example, resonance structures H-Xe^+^ :Cl^−^···HI, H-Xe^+^ Cl-H :I,^−^ and H-Xe^+^ Cl^∧^I were considered. Also note that the sum in this table is a maximum of 100%. The error in 100.1% is due to the weightings 74.3, 10.9, 14.9% of the resonance structures (H-Xe^+^ :Cl^−^, H:^−^ Xe^+^-Cl, H^∧^Cl) in HXeCl, where they are from 74.28, 10.85, 14.87%*.

Table 2 lists natural bond orders of the H-Xe and H^−^Y bonds in the monomer HXeY and its complex. It is worthwhile noting that the weighting and the corresponding bond order are equivalent for our studied cases in NBO/NRT framework if the former is expressed as a fraction rather than a percentage. For instance, the weightings of the H-Xe^+^:Cl^−^ in the monomer HXeCl and in the complex HXeCl···HCl are 74.3% and 71.8%, with corresponding bond order *b*_H−Xe_ 0.743 and 0.718, respectively. Generally, the larger the bond order is, the stronger the bond is. The data obtained from current NBO programs show the decrease of H-Xe bond orders, reflecting the weakening H-Xe bonds upon complexation. Obviously, these calculated results do not reflect the experimental fact: the strengthened H-Xe bond. This disagreement confirms that the H-Xe bond in HXeY is not a classical covalent bond.

**Table 2:** The bond orders (*b*) of H-Xe and H^∧^Y bonds for the monomer and their hydrogen-bonded complex, with the changes shown in parentheses.

**Monomers/complexes**	***b*_**H−Xe**_**	***b*_**H−Y**_**
HXeCl	0.743	0.149
HXeCl···H_2_O	0.705 (−0.038)	0.127 (−0.022)
HXeCl···HCl	0.718 (−0.025)	0.122 (−0.027)
HXeCl···HBr	0.723 (−0.020)	0.120 (−0.029)
HXeCl···HI	0.720 (−0.023)	0.121 (−0.028)
HXeCl···HCN	0.701 (−0.042)	0.131 (−0.018)
HXeCl···HCCH	0.764 (+0.021)	0.138 (−0.011)
HXeBr	0.713	0.171
HXeBr···H_2_O	0.675 (−0.038)	0.146 (−0.025)
HXeBr···HCl	0.682 (−0.031)	0.143 (−0.028)
HXeBr···HBr	0.687 (−0.026)	0.140 (−0.031)
HXeBr···HI	0.684 (−0.029)	0.141 (−0.030)
HXeBr···HCN	0.668 (−0.045)	0.150 (−0.021)
HXeBr···HCCH	0.646 (−0.067)	0.159 (−0.012)
HXeI	0.664	0.241
HXeI···H_2_O	0.616 (−0.048)	0.181 (−0.060)
HXeI···HCl	0.625 (−0.039)	0.200 (−0.041)
HXeI···HBr	0.631 (−0.033)	0.172 (−0.069)
HXeI···HI	0.628 (−0.036)	0.173 (0.068)
HXeI···HCN	0.616 (−0.048)	0.181 (−0.060)
HXeI···HCCH	0.591 (−0.073)	0.193 (−0.048)

This conclusion deserves some illustration. According to the natural bond orders' definition (Weinhold and Landis, 2005a), the H-Xe bond in HXeY is regarded as a classical covalent bond. The resonance structure H:^−^ Xe^+^-Y does not contribute to the H-Xe bond order at all. The H-Xe bond order is only contributed to by the resonance structure H-Xe^+^:Y^−^. Thus, the H-Xe bond order in Table 2 reflects merely the electron-sharing contribution in the H-Xe bond. It is insufficient to reflect the real strength between H and Xe in HXeY.

### H-Xe Bond in HXeY Is a CS Bond

The above studies on the H-Xe bond in HXeY confirm that it is not a classical covalent bond. Then, is it a CS bond? To address this question, we first need to solve the problem of the H-Xe bond order. Let us return to the original NBO/NRT theory. In the framework of NRT theory (Weinhold and Landis, 2005a), the H-Xe total bond strength in HXeY could be written in resonance-averaged form D(H-Xe) = ω_I_x D_I_ + ω_II_x D_II_, where “ω_I_” and “ω_II_” respectively correspond to the weighting of H-Xe^+^ Y^−^ and H:^−^ Xe^+^-Y; “D_I_” and “D_II_” represent the H-Xe bond strength in H-Xe^+^ Y^−^ and in H:^−^ Xe^+^-Y respectively. As we know, a bond order is roughly proportional to the bond strength or the bond length. If introducing two arbitrary constants is presumed to be expressed in terms of the same factor k, D_I_, and D_II_ can be written in the form, D_I_= k x *b*_I_ and D_II_= k x *b*_II_. Importantly, we obtain that *b*(H-Xe) = ω_I_x b_I_ + ω_II_x *b*_II_, where *b*_I_ represents the H-Xe bond order in resonance structure I, as we know, *b*_I_= 1; *b*_II_ refers to the H-Xe bond order in resonance structure II. Therefore, a calculation that includes two resonance structures is needed to deal with the H-Xe bond order. Note that the procedure employed to calculate the H-Xe CS bond order can be found in Supplementary Material “Explanation of the Procedure Employed to Calculate the BO of the H-Xe Bond.”

To obtain *b*_II_, we have to carry out some analyses on H:^−^ Xe^+^-Y. For this structure, where does the H-Xe bonding originate from? Here we emphasize that the H:^−^ Xe^+^-Y structure is a natural Lewis structure, in the NBO/NRT language. Consideration of the antibond of the Xe^+^-Y could lead to extension of the elementary Lewis structure concept to include hyperconjugative delocalization corrections in simple NBO perturbative estimates (Weinhold, 2012). Such hyperconjugative delocalization forms one starting point for our understanding of bonding about the H:^−^ Xe^+^-Y structure. As shown in Figure 3, one donor-acceptor interaction ${\text{n}}_{\text{H}}\to \sigma_{\text{Xe}-\text{Y}}^{*}$ exists in the H:^−^ Xe^+^-Y structure. On the basis of this result, we propose that the H-Xe bonding about the structure H:^−^ Xe^+^-Y is attributed to this donor-acceptor interaction. In our familiar language, it is dative bonding due to hyperconjunctive interaction.

However, the question was still left open: How do we estimate the degree of this H-Xe dative covalency? For this question, our research ideas are from the natural bond orders' definition proposed by Weinhold and Landis (2005b). If such a single dative bond order *b*_II_ is defined as 1, the NRT bond order of the dative structure is equal to the corresponding fractional weighting. Taking HXeCl as one illustrative example, the dative weightings of monomer HXeCl and hydrogen-bonded complex HXeCl**···**HCl are 10.9 and 14.6%, respectively. This simple method lets us respectively estimate the dative bond orders: 0.109 and 0.146.

The above discussion is an effort to rationalize *b*_II_. We now begin a discussion with a sum of ω_I_x *b*_I_, and ω_II_x *b*_II_. For convenience, we use *b*_H−Xe_ (electron-sharing) = ω_I_x *b*_I_,, *b*_H−Xe_ (dative) = ω_II_x *b*_II_. As shown in NRT theory, it is a sum of *b*_H−Xe_ (dative) and *b*_H−Xe_ (electron-sharing) that can reflect the H-Xe total strength in HXeY. Figure 4 shows correlation plots between the H-Xe bond order [*b*_H−Xe_ (dative) + *b*_H−Xe_ (electron-sharing) and the H-Xe bond length *R*_H−Xe_ for our studied Xe species. One additional example is also shown in Figure 4 for Kr analogs. Good correlation shown in Figure 4 for our studied species except Y = I, provides evidence to support our estimated method. Not enough good correlation in Y = I case may be due to a larger coupling effect between ${\text{n}}_{\text{Y}}\to o_{\text{H}-\text{Xe}}^{*}$ in HXeY and ${\text{n}}_{\text{Y}}\to o_{\text{H}-\text{X}}^{*}$ in its H-bonding complex.

On the basis of the data of the H-Xe bond order, we can analyze the H-Xe total bonding of HXeY. From preceding NRT analyses, we have shown that the electron-sharing bond orders [*b*_H−Xe_ (electron-sharing)] in the monomer and in the complex are 0.743 and 0.718, respectively, and 0.109 and 0.146 for the dative bond orders [*b*_H−Xe_ (dative)]. Thus, summing *b*_H−Xe_ (dative) to *b*_H−Xe_ (electron-sharing) yields 0.852 and 0.864 for the HXeCl monomer and the HXeCl···HCl complex, respectively. Obviously, the HXeCl complexing with the molecule HCl leads to an increase of the H-Xe bond order. The same is true for other complexes studied here. Table 3 lists the total bond order of the H-Xe bond in HXeY and in its hydrogen-bonded complexes. Note that it includes dative contribution and electron-sharing contribution of H-Xe bonding. As shown in Table 3, the HXeY complexing with the small molecule leads to an increase of the H-Xe bond order. In other words, it is an enhancement of the H-Xe bond. Such a result is consistent with experimental observations about our studied cases.

**Table 3:** The H-Xe bond orders: *b*_H−Xe_ (electron-sharing), *b*_H−Xe_ (dative), and the total [*b*_H−Xe_ (electron-sharing) + *b*_H−Xe_ (dative)], for the monomer and their hydrogen-bonded complex, with the changes shown in parentheses.

**Monomers/complexes**	***b*_**H−Xe**_ (electron-sharing)**	***b*_**H−Xe**_ (dative)**	***b*_**H−Xe**_ (total)**
HXeCl	0.743	0.109	0.852
HXeCl···H_2_O	0.705 (−0.038)	0.164 (+0.055)	0.869 (+0.017)
HXeCl···HCl	0.718 (−0.025)	0.156 (+0.047)	0.874 (+0.022)
HXeCl···HBr	0.723 (−0.020)	0.153 (+0.044)	0.876 (+0.024)
HXeCl···HI	0.720 (−0.023)	0.154 (+0.045)	0.874 (+0.022)
HXeCl···HCN	0.701 (−0.042)	0.169 (+0.060)	0.870 (+0.018)
HXeCl···HCCH	0.764 (+0.021)	0.097 (−0.012)	0.861 (+0.009)
HXeBr	0.713	0.116	0.829
HXeBr···H_2_O	0.675 (−0.038)	0.178 (+0.062)	0.853 (+0.024)
HXeBr···HCl	0.682 (−0.031)	0.172 (+0.056)	0.854 (+0.025)
HXeBr···HBr	0.687 (−0.026)	0.168 (+0.052)	0.855 (+0.026)
HXeBr···HI	0.684 (−0.029)	0.170 (+0.054)	0.854 (+0.025)
HXeBr···HCN	0.668 (−0.045)	0.182 (+0.066)	0.850 (+0.021)
HXeBr···HCCH	0.646 (−0.067)	0.194 (+0.078)	0.840 (+0.011)
HXeI	0.664	0.095	0.759
HXeI···H_2_O	0.616 (−0.048)	0.203 (+0.108)	0.819 (+0.060)
HXeI···HCl	0.625 (−0.039)	0.171 (+0.076)	0.796 (+0.037)
HXeI···HBr	0.631 (−0.033)	0.192 (+0.097)	0.823 (+0.064)
HXeI···HI	0.628 (−0.036)	0.192 (+0.097)	0.820 (+0.061)
HXeI···HCN	0.616 (−0.048)	0.203 (+0.108)	0.819 (+0.060)
HXeI···HCCH	0.591 (−0.073)	0.216 (+0.121)	0.807 (+0.048)

It becomes clear that the bonding between H and Xe in HXeY must meet two conditions. First, the resonance bonding between H^−^ Xe^+^-Y and H-Xe^+^ Y^−^ is a necessary condition. Second, for the H-Xe bond order, including contribution of these two resonance structures is essential. These two conditions are, in effect, consistent with emphases in original CS bonding concept paper (Shaik et al., 1992). The mixed covalent-ionic description, such as F· ·F↔F^−^ F^+^, is an essential feature of CS bonding, wherein most, if not the entire, bond energy is provided by the covalent-ionic resonance energy. And both the covalent and ionic structures must be treated explicitly and on an equal footing. Thus, we conclude that the H-Xe bond in HXeY is a charge-shift bond.

It should be noted, however, that neither the H-Xe bond length nor its bond strength can be reliable probes for addressing the question on whether the bond between H and Xe in HXeY should be classified as a CS bond or a classical covalent bond.

### H-Xe CS Bonding Character

Now that the H-Xe bond in HXeY is a charge-shift bond, a new and unique form bonding, one question which arises is whether the unusual H-Xe blue shift is its CS bonding character in IR spectroscopy.

Deep analyses on the data in Table 3 show that the complexation leads to an increase of *b*_H−Xe_ (dative) while *b*_H−Xe_ (electron-sharing) decreases, for most Xe cases. The competition of these two opposite factors results in the strengthened H-Xe bonds, corresponding to the blue shifts. Here, we want to point out that unlike other Xe complexes, the HXeCl**···**HCCH complex shows a difference in the dominant factor. It is the electron-sharing factor that dominates the H-Xe frequent shifts. The overall effect of two opposing factors is still an enhancement of the H-Xe bond and a blue shift for the H-Xe stretching frequency. These analyses reflect that the monomer-to-complex blue shifts of H-Xe stretching modes for HXeY species should be attributed to the balance of dative and electron-sharing covalency in H-Xe bonds, corresponding to H^−^ Xe^+^-Y and H-Xe^+^ Y^−^ resonance structures. In brief, the H-Xe frequent shifts in HXeY**···**H_2_O/HCl/HBr/HI/HCCH/HCN hydrogen-bonded complexes is controlled by a balance of two factors acting in opposite directions.

All in all, blue shifts of H-Xe vibrational frequencies are controlled by a balance of two opposing factors for dative and electron-sharing covalency. The blue shift in our studied complexes can be seen as a normal spectroscopic phenomenon. It is natural for us to conclude that the H-Xe blue shift is the H-Xe CS bonding character in IR spectroscopy.

## Summary and Conclusions

The H-Xe bonding in HXeY has been a debated question in the chemical community. The usual answer is that it is classically covalent in character, or to be exact, it is electron-sharing. The unusual blue shifts of complexes HXeY···HX (Y = Cl, Br, I; X = OH, Cl, Br, I, CN, CCH) definitely reflect the unusual features of H-Xe bonding in noble gas hydrides. Via observed blue-shifted phenomena, we have computationally investigated the H-Xe bonding in HXeY from an NBO/NRT perspective.

We establish that the resonance bonding between H-Xe^+^ Y^−^ and H^−^ Xe^+^-Y is an essential feature of the H-Xe bonding, and that the H-Xe bonding in these two resonance structures must be considered explicitly. Specifically, its bonding includes the ${\text{n}}_{\text{H}}\to \sigma_{\text{Xe}-\text{Y}}^{*}$ donor-acceptor interaction in H^−^ Xe^+^-Y, as well as the electron-sharing interaction in H-Xe^+^ Y^−^; the H-Xe bond order is contributed to by these two resonance structures. We confirm that the H-Xe bond in HXeY is not a classical covalent bond but a charge-shift bond, and that H-Xe blue shifts is a normal spectroscopic phenomenon.

Our conclusions are (1) the H-Xe bond in HXeY is a charge-shift bond. (2) The H-Xe blue shift in its hydrogen-complexes is its CS bonding character in IR spectroscopy.

The first conclusion is consistent with ab initio VB methods' insight into the F-Xe CS bonding in XeF_2_ (Braïda and Hiberty, 2013). But we note a little difference in the understanding of CS bonding mechanism. We stress the point that the H-Xe CS bonding is due to the resonance between H-Xe^+^:I^−^ and H:^−^ Xe^+^-I, based on the natural Lewis structures' concept, whereas Shaik et al. (1992) think that CS bonding is due to strong mixing between the covalent structure and the ionic structure, based on Pauling-type Lewis structures' concepts.

Finally, we want to point out that H-Xe CS bonding is significantly different from some two-structure resonance bonding, such as hydrogen-bonding, although there is a formal resemblance in their resonance description. We have noticed that the bond order of hydrogen-bonding in literature (Jiao and Weinhold, 2019) is only considered a contribution from one resonance structure; the other does not contribute to bond orders of hydrogen-bonding at all. Why is CS bonding not important in hydrogen-bonding? More studies are on the way to answer such a question and to generalize CS bonding models. We believe that more surprise will be gained via NBO/NRT methods, in particular, new-type NBO-based NRT methods on analyses for larger CS bonding species (Glendening et al., 2019).

## Data Availability Statement

The raw data supporting the conclusions of this article will be made available by the authors, without undue reservation, to any qualified researcher.

## Author Contributions

GZ, CS, and DC made contributions to the design of the study. YS, XZ, JS and LF performed the calculations. GZ and YS wrote the draft of the manuscript. All the authors contributed to the manuscript revision, read and approved the submitted version.

## Conflict of Interest

The authors declare that the research was conducted in the absence of any commercial or financial relationships that could be construed as a potential conflict of interest.
