# Supplementary material for: Charge-Shift Bonding in Xenon Hydrides: An NBO/NRT Investigation on HXeY···HX (Y = Cl, Br, I; X = OH, Cl, Br, I, CCH, CN) via H-Xe Blue-Shift Phenomena
Source: Front Chem. 2020 Apr 23;8:277. doi: 10.3389/fchem.2020.00277 (PMC7191121; doi:10.3389/fchem.2020.00277)
Supplement: Supplementary file 1 [file Data_Sheet_1.PDF]

Charge-Shift Bonding in Xenon Hydrides: An NBO/NRT Investigation on HXeY···HX (Y = Cl, Br, I; X = OH, Cl, Br, I, CCH, CN) via H-Xe Blue-Shift Phenomena

Supporting Information

Guiqiu Zhang,*[1] Yue Su,[1] Xiaoran Zou,[1] Lei Fu,[1] Junjie Song,[1] Dezhan Chen[1] and Chuanzhi Sun*[1]

Table S1. Calculated bond lengths $R_{H\text{-}Xe}$ and $R_{Xe\text{-}Y}$ (in Å) and monomer-to-complex frequency blue shifts (in cm^{-1}) of H-Xe stretching mode for hydrogen-bonded complexes HXeY ··· HX (Y = Cl, Br, I; X = OH, Cl, Br, I, CN, CCH) as well as the angle (in °) between HXeY and HX in Structure A at the MP2/def2-TZVPPD level of theory, with available experimental blue shifts. The data in parentheses were calculated at the CCSD(T) level of theory.

Monomers/Complexes	$R_{H\text{-}Xe}$	$R_{Xe\text{-}Y}$	Angle(°)	H-Xe blue shifts	H-Xe blue shifts (exp)
HXeCl	1.666	2.616			
HXeCl ··· H_2O	1.646	2.671	75	101 (118)[c]	82[c]
HXeCl ··· HCl	1.645	2.672	82	103	116[d]
HXeCl ··· HBr	1.644	2.674	82	102	
HXeCl ··· HI	1.646	2.670	82	114	
HXeCl ··· HCN	1.648	2.661	92	88	
HXeCl ··· HCCH	1.655	2.642	79	51	
HXeBr	1.679	2.774			
HXeBr ··· H_2O	1.656	2.825	71	116 (148)[c]	101[c]
HXeBr ··· HCl	1.657	2.822	77	110	122[d]
HXeBr ··· HBr	1.656	2.823	77	110	149[d]
HXeBr ··· HI	1.658	2.819	78	90	
HXeBr ··· HCN	1.658	2.817	88	106	
HXeBr ··· HCCH	1.667	2.798	75	58	
HXeI	1.708	2.976			
HXeI ··· H_2O	1.679	3.022	67	139 (188)[c]	138[c]
HXeI ··· HCl	1.682	3.017	72	122 (167)[b]	94, 111, 155[b]
HXeI ··· HBr	1.681	3.017	72	122 (149)[a]	110, 157[a]
HXeI ··· HI	1.683	3.012	72	107 (120)[a]	75, 96[a]
HXeI ··· HCN	1.680	3.015	83	132	
HXeI ··· HCCH	1.693	2.996	70	67 (104)[b]	49, 55[b]

[a] From ref. Tsuge et al., 2013.

[b] From ref. Zhu et al., 2015.

[c] From ref. Tsuge et al., 2014.

[d] From ref. Lignell et al., 2008.

Table S2. Second-order perturbation energies($E^{(2)}$, kcal ·mol^{-1}) due to donor-acceptor interactions in HXeY at the MP2/def2-TZVPPD level of theory.

Molecules	$n_Y \rightarrow \sigma^*_{H-Xe}$	$n_H \rightarrow \sigma^*_{Xe-Y}$	$n_{Xe} \rightarrow \hat{\sigma}^*_{H-Y}$
HXeCl	64.57	3957.00	1363.55
HXeBr	65.75	5187.90	1102.19
HXeI	70.75	5125.55	845.16

Explanation of the Procedure Employed to Calculate the BO of the Xe-H Bond

1. Conceptual Model in NBO/NRT

In the NBO/NRT framework, the fundamental starting point for a rational electronic theory of bonding is the Lewis-structure representation of the shared and unshared electrons in each atomic valence configuration, as formulated by Lewis. Based on the Lewis-structure model, perturbation theory is used to calculate systematically the corrections that bring the Lewis-structure model into an improved Lewis-structure model. In NBO/NRT language, it is the natural Lewis-structure model.

Subsequently, bonding analysis can be dissected into localized and delocalized contributions.

Take HXeY as an illustrative example. HXeY could be described as the hybrid of three resonance structures: H-Xe^{+} Y^{-}, H:$^{-}$ Xe^{+}-Y, and H^Y. When we focus on the H-Xe bond, the structure Ⅰ provides a localized contribution due to electron-sharing bonding. In structure Ⅱ, it is a delocalized contribution arising from a donor-acceptor interaction. In chemical language, it is dative bonding. Structure Ⅲ is a long-bonding structure. There is no contribution to the H-Xe bond strength, because there are neither localized contributions nor delocalized contributions to the H-Xe bonding.

2. Calculate the Weighting of Resonance Structure

A quantitative resonance theory can help us to find the weighting of resonance structure α, according to

$$D(\text{true}) = \sum_{\alpha} \omega\alpha D\alpha^{(\mathbf{L})}$$

where D(true) is the true density matrix of the system of interest. $D\alpha^{(L)}$ is corresponding to the density matrix of the resonance structure α. Note that $\sum_{\alpha} \omega\alpha = 1$.

3. Calculate Each Property of the System

In the NRT of framework, each property <P>true of the true delocalized system can be represented in resonance-averaged form

$$\langle P\rangle\text{true} = \sum_{\alpha} \omega\alpha < P > \alpha$$

where <P>α is the value of the property for natural resonance structure α.

Still take HXeY as an example. The H-Xe bond strength D(H-Xe) can be written as D(H-Xe) = $\omega_{I} D_{I} + \omega_{II} D_{II} + \omega_{III} D_{III}$.

From the conceptual analysis of the natural Lewis-structure, we know $D_{III} = 0$. Thus, D(H-Xe) = $\omega_{I} D_{I} + \omega_{II} D_{II}$

4. Calculate Bond Order of H-Xe

When using the bond order to reflect the H-Xe bond strength, D_{I} and D_{II} can be written as $D_{I} = k_{I} \omega_{I} b_{I}$, $D_{II} = k_{II} \omega_{II} b_{II}$, where b_{I} represents the normal covalent H-Xe bond order in resonance structure Ⅰ, b_{II} is the H-Xe dative bond order in resonance structure Ⅱ.

If $k_{I} = k_{II} = k$, and $b_{I} = b_{II} = 1$, we obtain b(H-Xe) = $\omega_{I} + \omega_{II}$ for our studied HXeY.

It is worthwhile noting that the present NBO/NRT methods could not provide the ratio of k_{I} / k_{II}, and that the adequacy in calculating the BO of the H-Xe bond has been tested through indirect comparisons with experimental results.
